# RUNX2 Phase Separation Mediates Long‐Range Regulation Between Osteoporosis‐Susceptibility Variant and *XCR1* to Promote Osteoblast Differentiation

**DOI:** 10.1002/advs.202413561

**Published:** 2024-12-20

**Authors:** Yan Zhang, Xin‐Hao Li, Pai Peng, Zi‐Han Qiu, Chen‐Xi Di, Xiao‐Feng Chen, Nai‐Ning Wang, Fei Chen, Yin‐Wei He, Zhong‐Bo Liu, Fan Zhao, Dong‐Li Zhu, Shan‐Shan Dong, Shou‐Ye Hu, Zhi Yang, Yi‐Ping Li, Yan Guo, Tie‐Lin Yang

**Affiliations:** ^1^ Key Laboratory of Biomedical Information Engineering of Ministry of Education Key Laboratory of Biology Multiomics and Diseases in Shaanxi Province Higher Education Institutions and Biomedical Informatics & Genomics Center School of Life Science and Technology Xi'an Jiaotong University Xi'an Shaanxi 710049 China; ^2^ Department of Orthopedics The First Affiliated Hospital of Xi'an Jiaotong University Xi'an Shaanxi 710061 China; ^3^ Key Laboratory of Shaanxi Province for Craniofacial Precision Medicine Research College of Stomatology Xi'an Jiaotong University Xi'an Shaanxi 710004 China; ^4^ Department of Joint Surgery Honghui Hospital Xi'an Jiaotong University Xi'an Shaanxi 710054 China; ^5^ Division in Cellular and Molecular Medicine Department of Pathology and Laboratory Medicine Tulane University School of Medicine Tulane University New Orleans LA 70112 USA

**Keywords:** GWAS, osteoporosis, phase separation, RUNX2, XCR1

## Abstract

GWASs have identified many loci associated with osteoporosis, but the underlying genetic regulatory mechanisms and the potential drug target need to be explored. Here, a new regulatory mechanism is found that a GWAS intergenic SNP (rs4683184) functions as an enhancer to influence the binding affinity of transcription factor RUNX2, whose phase separation can mediate the long‐range chromatin interaction between enhancer and target gene *XCR1* (a member of the GPCR family), leading to changes of *XCR1* expression and osteoblast differentiation. Bone‐targeting AAV of *Xcr1* can improve bone formation in osteoporosis mice, suggesting that *XCR1* can be a new susceptibility gene for osteoporosis. This study is the first to link non‐coding SNP with phase separation, providing a new insight into long‐range chromatin regulation mechanisms with susceptibility to complex diseases, and finding a potential target for the development of osteoporosis drugs and corresponding translational research.

## Graphical abstract

A GWAS intergenic SNP (rs4683184) acts as an enhancer, altering the binding affinity of the transcription factor RUNX2 and resulting in changes in XCR1 expression and osteoblast differentiation. RUNX2 phase separation mediates the long‐range regulation between rs4683184 and XCR1. Bone‐targeting AAV of XCR1 improves bone formation and increases bone mineral density in mice with osteoporosis.

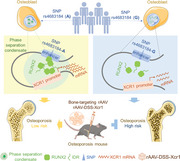

## 1 Introduction

Osteoporosis is an inevitable degenerative skeletal disease in elderly people characterized by low bone mineral density (BMD) with high heritability.^[^ ^1^, ^2^, ^3^, ^4^ ^]^ Previous genome‐wide association studies (GWASs) have successfully identified multiple loci associated with BMD and osteoporosis.^[^ ^5^ ^]^ Some GWAS genes have been used as drug targets to treat osteoporosis, and successfully have entered clinical trials or have been marketed, such as *Calcium sensing receptor, Dickkopf‐related protein 1, Sclerostin, and Receptor activator of nuclear factor kappa B ligand*.^[^ ^4^, ^6^ ^]^ However, most of the loci identified by GWASs are located in non‐coding regions, which poses great challenges to the translation of GWAS findings into clinical applications.

G protein‐coupled receptors (GPCRs) are important and necessary for regulating osteoblast differentiation and bone remodeling, and related genes of GPCR family could be the pivotal targets for the clinical treatment of osteoporosis.^[^ ^7^, ^8^ ^]^ Generally, drug targets with genetic support are twice as likely to succeed in clinical trials.^[^ ^9^ ^]^ Specifically, GWASs have identified some non‐coding susceptibility single nucleotide polymorphisms (SNPs) located near X‐C motif chemokine receptor 1 (*XCR1*), which is associated with BMD. XCR1, also known as G protein‐coupled receptor 5 (GPR5), is a vital member of the GPCR family.^[^ ^10^ ^]^ We hypothesize that *XCR1* might be a new potential target for osteoporosis, but the functional roles between GWAS SNPs and *XCR1* underlying the pathological mechanism of osteoporosis remain to be explored.

Non‐coding SNPs often reside in *cis*‐regulatory elements, such as enhancers, where they recruit transcription factors (TFs) to activate target genes expression through long‐range chromatin interactions.^[^ ^11^, ^12^, ^13^ ^]^ It has been proposed that TFs may be responsible for shaping 3D genome architecture,^[^ ^14^, ^15^ ^]^ but the inner mechanisms remain incompletely understood. Recently, liquid‐liquid phase separation (LLPS) has been recognized as playing a key role in transcriptional regulation.^[^ ^16^, ^17^, ^18^, ^19^ ^]^ Intrinsically disordered regions (IDR) of TFs enrich the transcriptional device into specific enhancers and drive chromatin looping through phase separation, further achieving the compartmentalization of the transcription process and regulating the expression of target genes.^[^ ^16^, ^20^, ^21^ ^]^ Since different alleles of SNPs could influence the binding affinity of TFs, we speculate that the long‐range regulation between non‐coding SNPs and target gene might be mediated by phase separation.

Therefore, in this study, we elucidate the functional basis that a GWAS intergenic SNP (rs4683184) acts as an enhancer, which alters the binding affinity of RUNX family transcription factor 2 (RUNX2) and leads to differential *XCR1* expression and osteoblast differentiation. Importantly, we discover that RUNX2 phase separation could mediate the long‐range regulation between rs4683184 and *XCR1*. *XCR1* could promote osteoblast differentiation. Bone‐targeting adeno‐associated virus (AAV) carrying the *Xcr1* gene could improve bone formation and increase BMD in osteoporosis mice. Together, our findings not only clarify a new regulatory mechanism by which transcription factor and non‐coding SNP affect disease susceptibility through phase separation, but also provide a new target for the development of osteoporosis drugs.

## 2 Results

### 2.1 XCR1 Significantly Promotes Osteoblast Differentiation

**Figure 1:**
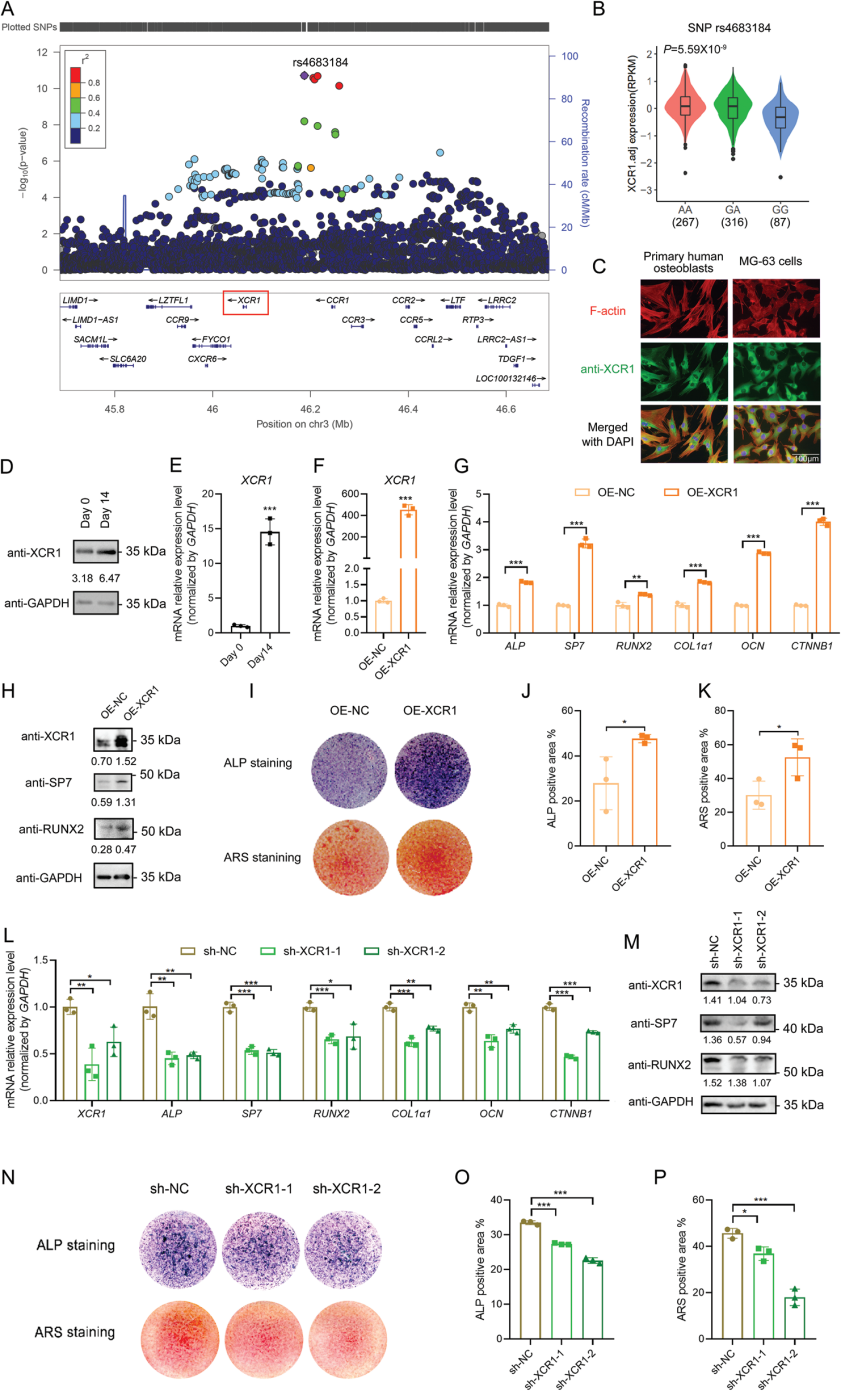
X‐C motif chemokine receptor 1 (XCR1) significantly promoted osteoblast differentiation. A) Genome‐wide association study (GWAS) identified susceptibility single nucleotide polymorphisms (SNPs) around *XCR1* associated with human bone mineral density (BMD). B) Violin plot for *XCR1* expression in human whole blood samples with different genotypes of SNP rs4683184 from GTEx V8 (https://www.gtexportal.org/home/). Samples sizes of human whole blood: n (AA) = 267, n (GA) = 316, n (GG) = 87. C) Immunofluorescence (IF) staining of XCR1 in primary human osteoblasts and human osteoblastic line MG‐63. Bar: 100 µm. D) Western blot of XCR1 in MG‐63 cells induced osteoblast differentiation for 14 days. E) RT‐qPCR was used to explore the *XCR1* mRNA expression level in induced MG‐63 cells on day 0 and day 14. F,G) *XCR1* expression and osteoblast differentiation marker genes *Alkaline phosphatase (ALP), Sp7 transcription factor (SP7), RUNX family transcription factor 2 (RUNX2), Collagen type I alpha 1 chain (COL1α1), Osteocalcin (OCN), and Catenin beta 1 (CTNNB1)* expressions were detected by RT‐qPCR in control (OE‐NC) and *XCR1* overexpression (OE‐XCR1) MG‐63 cells. H) Western blot of XCR1, SP7, and RUNX2 in MG‐63 cells. I) ALP staining and Alizarin red S (ARS) staining in OE‐NC and OE‐XCR1 group cells with induced 14 days. J,K) Quantification of I. L,M) RT‐qPCR and western blot were executed to test the expression of XCR1 and osteoblast differentiation marker genes in control (sh‐NC) and *XCR1* knock down (sh‐XCR1) MG‐63 cells. N) ALP staining and ARS staining in sh‐NC and sh‐XCR1 group MG‐63 cells with induced 14 days. O,P) Quantification of N. Values of *P* was determined with a two‐tailed t‐test. *P < 0.05, **P < 0.01, ***P < 0.001.

GWAS has identified one conditionally independent association signal (SNP rs4683184) for human heel ultrasounds estimated bone mineral density (eBMD) near the *XCR1* gene (**Figure** **1**A, **Table** **1**). Cis‐eQTL analysis showed that the osteoporosis‐susceptibility SNP rs4683184‐A allele was significantly associated with increased expression of *XCR1* (*P* = 5.59 × 10^−9^, Figure 1B). Immunofluorescence (IF) staining demonstrated that XCR1 was widely expressed in primary human osteoblasts and human osteoblastic cell line MG‐63 (Figure 1C; Figure S1A, Supporting Information). These findings suggested that *XCR1* might be a new susceptibility gene for osteoporosis. However, the precise function of *XCR1* in osteoporosis and osteoblasts remained unknown. To explore the effect of *XCR1* on osteoblasts, we induced osteoblast differentiation and observed that XCR1 expression was increased in the induced osteoblasts (Figure 1D,E; Figure S1B,C, Supporting Information). Overexpression of *XCR1* significantly promoted the mRNA expression and protein accumulation of osteoblast differentiation marker genes (Figure 1F–H), and enhanced Alkaline phosphatase (ALP) activity and mineralized nodule formation by ALP staining and Alizarin Red S (ARS) staining in MG‐63 cells (Figure 1I–K). Similarly, knockdown of *XCR1* expression in MG‐63 cells distinctly reduced the expression of osteoblast differentiation marker genes (Figure 1L,M), as well as ALP activity and mineralized nodule formation (Figure 1N–P). We also tested the effect of *XCR1* on osteoblast proliferation using the Cell counting kit‐8 experiment, but found no significant difference in cell proliferation levels compared with the control group (Figure S1D, Supporting Information). Together, these results indicate the important role of *XCR1* in osteoblast differentiation.

**Table 1:** Conditional estimated bone mineral density (eBMD) GWAS results of top 9 SNPs.

No.	SNP	Physical position	Genic position	A1/A2^a)^	MAF^b)^	Heel BMD	
						*β* ^c)^	P
1	rs4683184	46187707	Intergenic^d)^	G/A	0.361182	−0.01289	1.93 × 10^−11^
2	rs2172247	46214670	Intergenic	T/C	0.361351	−0.01288	2.03 × 10^−11^
3	rs7616215	46205686	Intergenic	C/T	0.36151	−0.01281	2.56 × 10^−11^
4	rs2087726	46208310	Intergenic	G/A	0.361515	−0.01275	3.18 × 10^−11^
5	rs2201150	46258902	Intergenic	T/C	0.363474	−0.01252	7.08 × 10^−11^
6	rs7648466	46187522	Intergenic	A/G	0.281207	−0.01193	6.28 × 10^−9^
7	rs2172246	46214685	Intergenic	C/A	0.281542	−0.01171	1.16 × 10^−8^
8	rs1491961	46250348	Upstream^e)^	T/C	0.283387	−0.01141	2.53 × 10^−8^
9	rs3181077	46250652	Upstream	C/T	0.283549	−0.0113	3.38 × 10^−8^

^a)^ A1 represents the minor allele for each single nucleotide polymorphism (SNP).

^b)^ MAF refers to the minor allele frequency in the discovery sample.

^c)^ *β* is calculated with respect to the minor allele.

^d)^ Intergenic is a region between two genes. If a SNP is not in the coding region, intron, UTR (untranslated region), or upstream and downstream region of any gene, it is considered to be in the gene intergenic region.

^e)^ Upstream refers to the overlapping 1 kb (1000 base pairs) region upstream of the gene transcription start site, as annotated by ANNOVAR.^[^ ^40^ ^]^

### 2.2 SNP rs4683184 Acts as an Enhancer to Regulate *XCR1* Expression via Long‐Range Loop in Osteoblasts

**Figure 2:**
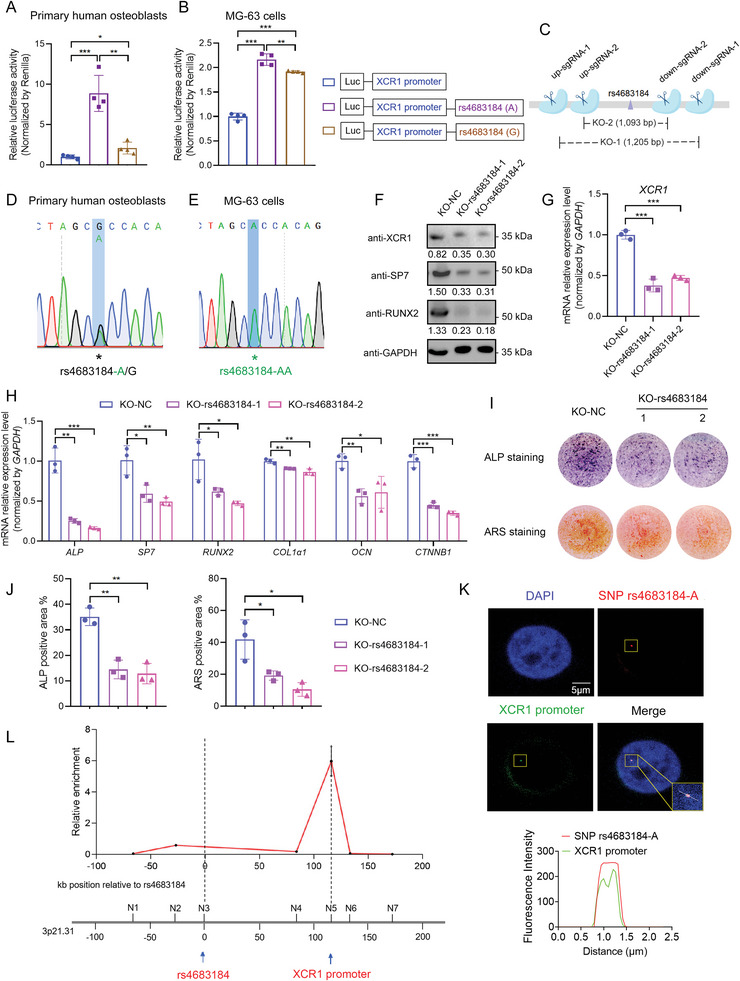
Non‐coding SNP rs4683184 remotely regulated the expression of target gene *XCR1* in osteoblast differentiation. A,B) Dual luciferase reporter assay for *XCR1* promoter containing the region surrounding either rs4683184‐A/‐G, or individual *XCR1* promoter in primary human osteoblasts and MG‐63 cells, respectively. Luciferase signals were normalized to Renilla activity. C) The scheme for deleting the region containing rs4683184 by CRISPR/Cas9. D) The genotype of rs4683184 in human primary osteoblast. E) The genotype of rs4683184 in human osteoblast‐like MG‐63 cell line. F) Western blot of XCR1, SP7, and RUNX2 protein expression in control (KO‐NC) and in rs4683184 knockout (KO‐rs4683184) MG‐63 cells. G,H) mRNA expression levels of *XCR1* and osteoblast differentiation marker genes *ALP*, *SP7*, *RUNX2*, *COL1α1*, *OCN* and *CTNNB1* were detected by RT‐qPCR in MG‐63 cells. I) ALP staining and ARS staining in MG‐63 cells were induced for 14 days. J) Quantification of I. K) 3D‐DNA fluorescence in situ hybridization (3D‐DNA FISH) experiments in MG‐63 cells. Plot of fluorescence intensity along the white line from left to right in merged image. Red: the DNA probes containing SNP rs4683184. Green: *XCR1* promoter DNA probes. Bar: 5 µm. L) Chromosome conformation capture (3C) in MG‐63 cells. The normalized relative enrichment of interaction between rs4683184 region (N3) and *XCR1* promoter region (N5) or other five sites (N1, N2, N4, N6, and N7) are shown. Values of *P* were determined with a two‐tailed t‐test. *P < 0.05, **P < 0.01, ***P < 0.001.

GWAS SNPs located near *XCR1* are found in the noncoding region with unknown functions. To explore their regulatory role on *XCR1*, we performed a series of experiments. We first screened susceptibility SNPs at 3p21.31 and found that SNP rs4683184 was the top associated SNP (*P* = 1.93 × 10^−11^) with heel BMD for the conditionally independent GWAS signal near *XCR1* (Table 1). The A allele of rs4683184 is associated with higher BMD (Table 1). Despite the ≈116 kb distance between rs4683184 and the *XCR1* gene in the genome, the rs4683184‐A allele was significantly associated with increased expression of *XCR1* (Figure 1B), and *XCR1* had the dominant expression level compared with nearby genes C‐C motif chemokine receptor 1 (CCR1) and C‐C motif chemokine receptor 3 (CCR3) in MG‐63 cells (Figure S2A, Supporting Information), suggesting the potential long‐range regulation between this SNP and *XCR1*. Further, rs4683184 was located within potential enhancer elements marked by strong active epigenetic marks (H3K27ac and H3K4me3) in human osteoblasts (Figure S2B, Supporting Information). To detect the enhancer effect of genomic fragments containing the major or minor allele of rs4683184 on *XCR1* expression, we used dual‐luciferase reporter assays in primary human osteoblasts and MG‐63 cells. We found that both alleles of rs4683184 could reinforce the promoter activity of *XCR1* (**Figure** **2**A,B). In particular, the rs4683184‐A allele had a significantly greater enhancing effect on XCR1 expression compared with the G allele, which is consistent with the eQTL analysis results (Figure 1B; Tables S1 and S2, Supporting Information).

To directly validate the enhanced regulation of rs4683184 on *XCR1*, we utilized CRISPR‐Cas9 technology to delete the region surrounding rs4683184 specifically in osteoblasts (Figure 2C). Upon screening the genotype of this SNP across various osteoblastic cell types, we found that MG‐63 osteoblastic cells exhibit a homozygous AA genotype for rs4683184, whereas hFOB1.19 osteoblastic cells are homozygous GG, and human primary osteoblasts are heterozygous A/G (Figure 2D,E; Figure S2C, Supporting Information). To investigate the impact of the rs4683184‐AA deletion on the regulation of *XCR1*, we chose to perform the CRISPR experiment in MG‐63 osteoblastic cells. The CRISPR deletion efficiency was validated by gel electrophoresis experiments, sequencing, and TIDE analysis (Figure S3A–D, Supporting Information). Compared with control cells, both protein and mRNA expression levels of XCR1 were significantly decreased in the SNP knockout (KO‐rs4683184) cells (Figure 2F,G; Figure S2F, Supporting Information), whereas we did not detect significant changes in the expression of *CCR1* and *CCR3* in KO‐rs4683184 cells (Figure S2D, Supporting Information), indicating that *XCR1* was the target gene of rs4683184. Importantly, the expression levels of osteoblast differentiation marker genes ALP, Sp7 transcription factor (SP7), RUNX2, Collagen type I alpha 1 chain (COL1α1), Osteocalcin (OCN), and Catenin beta 1 (CTNNB1) were significantly decreased in KO‐rs4683184 cells (Figure 2F,H; Figure S2F, Supporting Information). ALP and ARS staining showed that loss of rs4683184 significantly inhibited osteoblast differentiation activity and mineralized nodule formation (Figure 2I,J).

Besides, to confirm the long‐range chromatin interactions between rs4683184 and *XCR1* promoter, we performed chromosome conformation capture (3C) and 3D‐DNA fluorescence in situ hybridization (3D‐DNA FISH) experiments in MG‐63 cells. 3D‐DNA FISH showed that the probes containing rs4683184 and *XCR1* promoter probes had co‐localization in MG‐63 cells (Figure 2K). Our 3C experiment detected consistent results that the region where the SNP was located had remote enhancer regulation for *XCR1* promoter (Figure 2L). Taken together, these results provide direct evidence of long‐range enhancer‐promoter regulation between the rs4683184 and target gene *XCR1* in osteoblasts.

### 2.3 Transcription Factor RUNX2 Preferentially Binds to rs4683184‐A to Regulate *XCR1* Expression

**Figure 3:**
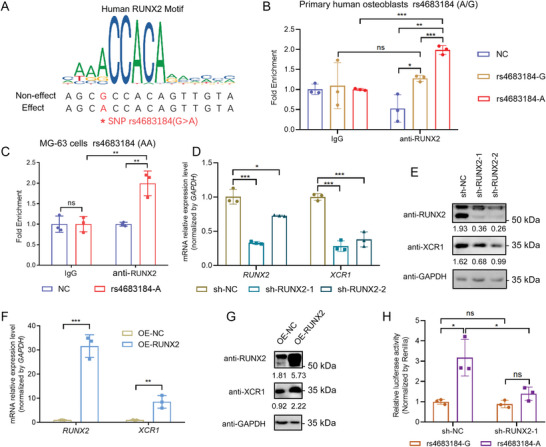
SNP rs4683184 specifically bound the transcription factor RUNX2 to remotely regulate *XCR1* expression in osteoblasts. A) The binding sequences of RUNX2 motif containing SNP rs4683184. B) Chromatin immunoprecipitation (ChIP) qPCR assay detected the binding between RUNX2 and rs4683184 (A/G) in human primary osteoblast. C) ChIP qPCR detected the binding between RUNX2 and rs4683184‐AA in MG‐63 cells. D,E) RT‐qPCR and western blot were implemented to validate the expression of RUNX2 and XCR1 in control (sh‐NC) and *RUNX2* knock down (sh‐RUNX2) MG‐63 cells. F,G) RT‐qPCR and western blot were used to examine the expression of RUNX2 and XCR1 in control (OE‐NC) and *RUNX2* overexpression (OE‐RUNX2) MG‐63 cells. H) Dual luciferase reporter assay for *XCR1* promoter containing the region surrounding either rs4683184‐A/‐G in control (sh‐NC) and RUNX2 knock down (sh‐RUNX2) cells. Luciferase signals were normalized to Renilla activity. Values of *P* were determined with a two‐tailed t‐test. ns. no significant difference. *P < 0.05, **P < 0.01, ***P < 0.001.

Regulation between non‐coding SNP enhancer and the target gene is usually mediated by transcription factors. The transcription factor motif analysis indicated that RUNX2, a key TF involved in osteoblast differentiation and osteoporosis,^[^ ^22^ ^]^ could preferentially bind to rs4683184‐A (**Figure** **3**A). To experimentally verify the motif prediction and to better elucidate the relationship between different genotypes of SNP rs4683184 and RUNX2, we performed ChIP‐qPCR in primary human osteoblasts with a heterozygous genotype (A/G) for SNP rs4683184. This revealed that the A allele exhibits a stronger binding affinity for RUNX2 compared to the G allele (Figures 2D,3B). We also conducted ChIP‐qPCR in MG‐63 cells and found a significant enrichment of RUNX2 binding to rs4683184‐A region with a homozygous genotype (A/A) for SNP rs4683184 (Figures 2E,3C). Further, we suppressed RUNX2 expression by shRNA and detected a significant reduction in XCR1 expression in MG‐63 cells (Figure 3D,E). Conversely, overexpression of RUNX2 in MG‐63 cells significantly enhanced both mRNA and protein expression levels of XCR1 (Figure 3F,G). We also found that the enhancement of XCR1 promoter activity by the rs4683184‐A was distinctly reduced when RUNX2 expression was inhibited, as determined by dual luciferase reporter gene assay (Figure 3H). Taken together, our results demonstrate that RUNX2 preferentially binds to rs4683184‐A to increase the enhancer function of regulating *XCR1* expression in osteoblasts.

### 2.4 RUNX2 Phase Separation Could Mediate the Long‐Range Regulation Between rs4683184 and *XCR1*

**Figure 4:**
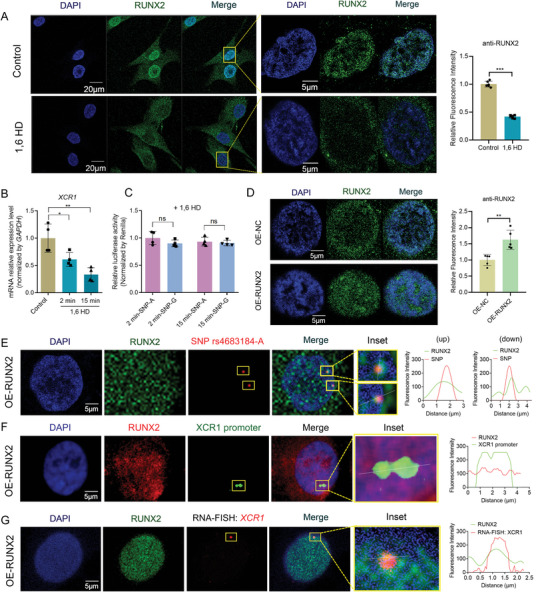
RUNX2 phase separation mediated the remote interaction between SNP rs4683184 and *XCR1*. A) Immunofluorescence (IF) staining and quantification analysis of RUNX2 in MG‐63 cells. 1.5% 1,6‐hexanediol (1,6 HD) was used to disturb the formation of phase separation condensates in cells. B) RT‐qPCR validated mRNA expression of *XCR1* in MG‐63 cells with 1.5% 1,6 HD treatment. C) Dual luciferase reporter assay for *XCR1* promoter containing the region surrounding rs4683184‐A/‐G in MG‐63 cells with 1.5% 1,6 HD treatment. Luciferase signals were normalized to Renilla activity. D) IF staining and quantification analysis of RUNX2 showed the presence of numerous RUNX2 condensates in the RUNX2 overexpression (OE‐RUNX2) group, when compared to the control (OE‐NC) MG‐63 cells. Bar: 5 µm. E) IF‐DNA FISH results revealed that the probe binding to SNP rs4683184‐A was localized on condensates of RUNX2 phase separation in OE‐RUNX2 MG‐63 cells. Plot of fluorescence intensity along the white line in merged image. Red: DNA probes containing SNP rs4683184. Green: anti‐RUNX2. Bar: 5 µm. F) The *XCR1* promoter was localized on condensates of RUNX2 phase separation in OE‐RUNX2 cells by IF‐DNA FISH. Plot of fluorescence intensity along the white line in merged image. Red: anti‐RUNX2. Green: DNA probes of *XCR1* promoter. Bar: 5 µm. G) IF‐RNA FISH results showed that the nascent *XCR1* RNA was localized on condensates of RUNX2 phase separation in OE‐RUNX2 cells. Plot of fluorescence intensity along the white line in merged image. Red: intron RNA probes of *XCR1*. Green: anti‐RUNX2. Bar: 5 µm. Values of *P* were determined with a two‐tailed t‐test. ns. no significant difference. *P < 0.05, **P < 0.01.

Recent studies have shown that TFs activate genes expression through their phase‐separation capacity, which can enrich transcriptional apparatus at enhancer regions.^[^ ^16^, ^20^, ^21^ ^]^ RUNX2 has the IDR region and can undergo phase separation in cells to participate in the regulation of transcriptional activity.^[^ ^23^ ^]^ However, the regulatory mechanism of RUNX2 phase separation in osteoblasts remains unclear. To deeply investigate whether RUNX2 phase separation mediates the remote regulation of rs4683184 and *XCR1*, 1,6‐hexanediol (1,6 HD) was used to disturb the formation of phase separation condensates in osteoblasts. Our results showed that the use of 1,6 HD inhibited the phase separation of RUNX2 in MG‐63 cells (**Figure** **4**A), and the mRNA expression of *XCR1* was significantly decreased after 1,6 HD treatment (Figure 4B). Further, we performed dual‐luciferase reporter assays in 1,6 HD treatment cells and found that rs4683184‐A lost its promoter enhancement effect on *XCR1* by comparing it with rs4683184‐G (Figure 4C). IF staining of RUNX2 demonstrated the presence of numerous RUNX2 condensates in the RUNX2 overexpression (OE‐RUNX2) group, suggesting that overexpression of RUNX2 promotes the formation of phase separation condensates in MG‐63 cells (Figure 4D). IF‐DNA FISH results revealed that the probes binding to SNP rs4683184‐A and the *XCR1* promoter were localized on condensates of RUNX2 phase separation in RUNX2 overexpression cells (Figure 4E,F). IF‐RNA FISH results confirmed that RUNX2 phase separation was involved in the regulation of the nascent *XCR1* transcription process by using intron RNA probes of *XCR1* (Figure 4G).

**Figure 5:**
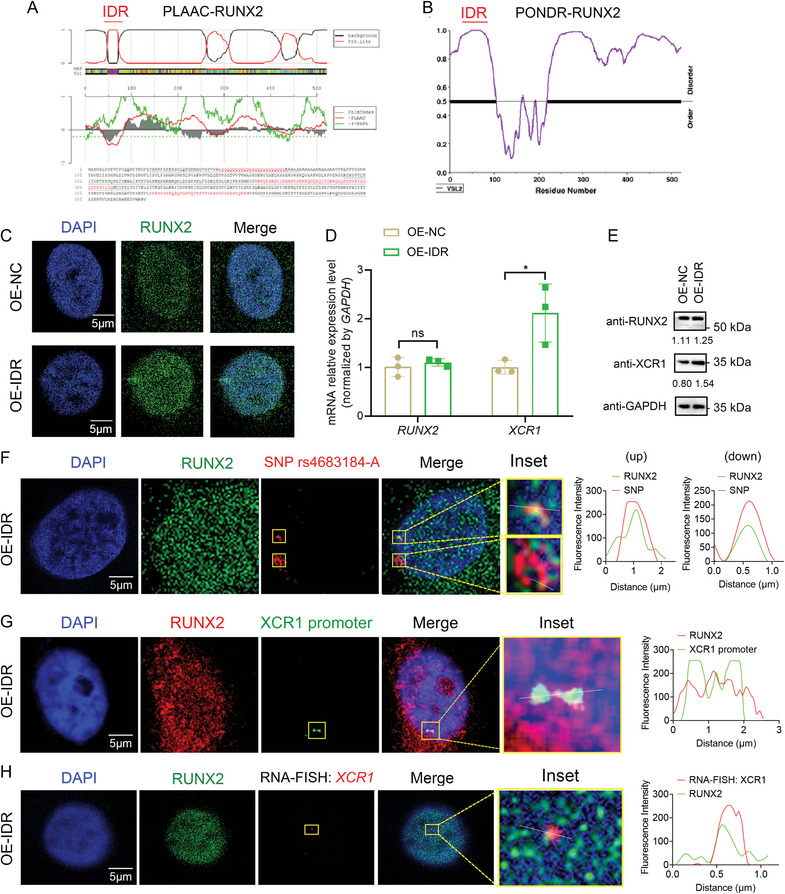
RUNX2‐IDR phase separation affected the transcriptional regulation between SNP rs4683184 and *XCR1* in osteoblasts. A,B) The intrinsically disordered region (IDR) sequence of RUNX2 in PLAAC and PONDER. C) IF staining of RUNX2 in MG‐63 cells for control (OE‐NC) and predicted IDR overexpression (OE‐IDR) groups. Bar: 5 µm. D,E) RT‐qPCR and western blot were used to examine the expression of RUNX2 and XCR1 in MG‐63 cells. F) IF‐DNA FISH results revealed that the probe binding to SNP rs4683184‐A was localized on condensates of RUNX2 phase separation in OE‐IDR MG‐63 cells. Plot of fluorescence intensity along the white line in merged image. Red: DNA probes containing SNP rs4683184. Green: anti‐RUNX2. Bar: 5 µm. G) The *XCR1* promoter was localized on condensates of RUNX2 phase separation in OE‐IDR cells by IF‐DNA FISH. Plot of fluorescence intensity along the white line in merged image. Red: anti‐RUNX2. Green: DNA probes containing *XCR1* promoter. Bar: 5 µm. H) IF‐RNA FISH results showed that the nascent *XCR1* RNA was localized on condensates of RUNX2 phase separation in OE‐IDR MG‐63 cells. Plot of fluorescence intensity along the white line in merged image. Red: intron RNA probes of *XCR1*. Green: anti‐RUNX2. Bar: 5 µm. Values of *P* were determined with a two‐tailed t‐test. ns. no significant difference. *P < 0.05.

To further explore the mechanism by which RUNX2 phase separation is involved in long‐range transcriptional regulation in osteoblasts, we analyzed the IDR sequence of RUNX2 in PLAAC and PONDER (**Figure** **5**A,B), and constructed IDR overexpression (OE‐IDR) MG‐63 cells. We found that the formation of RUNX2 phase separation condensates increased (Figure 5C), and both the protein and mRNA expression levels of XCR1were visibly lifted, whereas the expression of RUNX2 did not change significantly in OE‐IDR osteoblasts (Figure 5D,E). Furthermore, IF‐DNA FISH and IF‐RNA FISH results showed that SNP rs4683184, *XCR1* promoter, and nascent *XCR1* RNA were co‐localized with the RUNX2‐IDR phase separation condensates, respectively (Figure 5F–H). Collectively, these results suggest that RUNX2‐IDR phase separation plays an important role in mediating the long‐range transcriptional regulation between the rs4683184 enhancer and the target gene *XCR1* in osteoblasts.

### 2.5 AAV‐DSS‐Xcr1 Improves Bone Formation in Osteoporosis Mice

**Figure 6:**
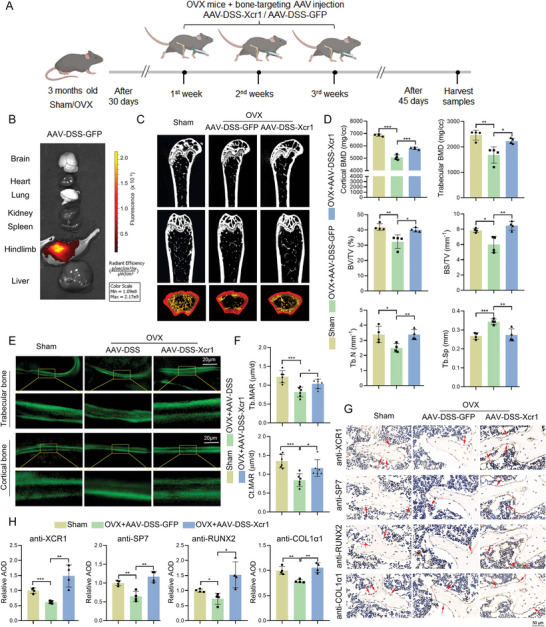
AAV‐DSS‐Xcr1 improved bone formation in osteoporosis mice. A) Schematic display of OVX surgery‐induced osteoporosis and subsequent AAV‐DSS‐Xcr1 treatment workflow. B) GFP expression in the hindlimb of AAV‐DSS‐GFP treatment mice. C,D) Micro‐CT analysis of microstructural bone parameters of the distal femurs including cortical bone BMD, trabecular bone BMD, Tb.N (trabecular number), BV/TV (bone volume/total tissue volume), BS/TV (bone surface/total tissue volume), Tb.Sp (trabecular separation) in different treatment mice group. E) Calcein double labeling images showing bone formation capacity. F) Mineral apposition rate (MAR) of trabecular bone and cortical bone. G) IHC staining of XCR1, SP7, RUNX2, and COL1α1 for mice femurs. H) Quantification analysis of G. The average optical density (AOD) analysis of IHC staining was performed using ImageJ. AOD is ratio of the integral optical density (IOD) to the area. Values of *P* were determined with a two‐tailed t‐test. *P < 0.05, **P < 0.01, ***P < 0.001.

To evaluate the role of *Xcr1* in osteoporosis in vivo, we performed ovariectomy (OVX) surgery in 3 months old female wild‐type (WT) mice, and utilized bone targeting AAV to overexpress *Xcr1* (AAV‐DSS‐Xcr1) by intra‐articular injection (**Figure** **6**A). We observed the GFP expression in the hindlimb of AAV‐DSS‐GFP treatment mice, but not in brain, heart, lung, kidney, spleen, and liver tissues, indicating successful bone targeting AAV infiltration (Figure 6B). Furthermore, immunohistochemistry (IHC) staining results showed that the expression of XCR1 in the AAV‐DSS‐Xcr1 group was significantly higher than that in the AAV‐DSS‐GFP group, confirming the overexpression efficiency of AAV‐DSS‐Xcr1 (Figure 6G,H). Micro‐CT results showed that cortical bone BMD, trabecular bone BMD, Tb.N (trabecular number), BV/TV (bone volume/total tissue volume), and BS/TV (bone surface/total tissue volume) decreased significantly in the OVX group by comparing with sham group, suggesting that our osteoporosis mice model was successfully constructed (Figure 6C,D). Further, we found that targeted overexpression of *Xcr1* in bone improved cortical bone BMD, trabecular bone BMD, Tb.N, BV/TV, and BS/TV, while reducing Tb.Sp (trabecular separation) in mice with osteoporosis (Figure 6C,D). Moreover, after OVX surgery, the mineral apposition rate (MAR, bone formation‐related parameter) of trabecular bone and cortical bone in the AAV‐DSS‐Xcr1 treatment group was higher than that in AAV‐DSS‐GFP treatment group (Figure 6E,F). Importantly, IHC staining showed that osteogenic marker protein expression (SP7, RUNX2, and COL1α1) of AAV‐DSS‐Xcr1 mice were obviously upregulated by comparing with AAV‐DSS‐GFP treatment group mice after OVX surgery (Figure 6G,H). Together, our results demonstrate that bone targeting overexpression of *Xcr1* could protect against bone loss in the OVX mice model, and *Xcr1* could be an effective target for osteoporosis treatment in vivo.

## 3 Discussion

**Figure 7:**
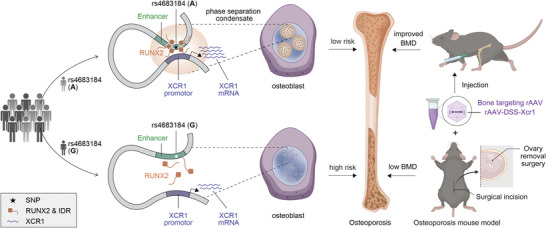
Proposed long‐range regulation model between osteoporosis susceptibility SNP rs4683184 and *XCR1* to promote osteoblast differentiation mediated by RUNX2 phase separation.

While GWASs have identified many loci associated with osteoporosis, the underlying genetic regulatory mechanisms and potential drug targets remain to be explored. In our study, we decipher a new regulatory mechanism that a GWAS intergenic SNP (rs4683184) functions as an enhancer to influence the binding affinity of RUNX2, whose phase separation could mediate the long‐range chromatin interaction between enhancer and target gene *XCR1*, leading to changes of *XCR1* expression and osteoblast differentiation. Bone‐targeting AAV of *Xcr1* could improve bone formation and increase BMD in mice with osteoporosis, suggesting that *XCR1* could be a new susceptibility gene for osteoporosis (**Figure** **7**). Our study provides a new perspective to understand the mechanism of TFs and non‐coding enhancer SNPs and finds a potential target for the development of osteoporosis drugs and corresponding translational research.

Notably, RUNX2 has the IDR region and can undergo phase separation in cells to participate in the regulation of transcriptional activity.^[^ ^23^ ^]^ Recently, the study of TF phase separation has provided the possibility to elucidate the mechanism of complex transcriptional spatial changes.^[^ ^13^ ^]^ Richard Young et al. found that transcriptional co‐activators can recruit phase separation droplets and enrich the transcription machine at the super‐enhancer, further achieving compartmentalization reactions during the transcription process.^[^ ^16^, ^24^ ^]^ Whole‐genome sequencing results of endogenous DNA‐associated disordered proteins indicate that a large number of peaks are enriched in disordered regions of TFs, and these IDRs of TFs could regulate long‐range chromatin interactions and be involved in the pathological process of disease.^[^ ^20^ ^]^ These studies provide ideas for elucidating the function of TFs in diseases related to transcriptional dysregulation.^[^ ^16^, ^17^, ^18^ ^]^ RUNX2 is a master TF in maintaining osteoblast differentiation and bone remodeling and regulates the downstream marker genes expression of osteoblast differentiation, such as Osterix/SP7, OCN, Osteopontin, and Bone Sialoprotein.^[^ ^22^, ^25^ ^]^ The absence of RUNX2 leads to a lack of mature osteoblasts and incomplete bone mineralization, resulting in clavicular hypoplasia and failure of cranial suture closure.^[^ ^25^ ^]^ Unfortunately, the in‐depth regulatory mechanism of RUNX2 in osteoblast differentiation remains unclear. Here, we found that RUNX2‐IDR obviously increased the formation of phase separation condensates and XCR1 expression in osteoblasts, and SNP rs4683184 specifically bound the RUNX2 in osteoblasts. Moreover, SNP rs4683184 and nascent RNA of *XCR1* were co‐localized with the RUNX2‐IDR phase separation condensate. Our results indicated that RUNX2 phase separation could mediate the remote regulation between SNP rs4683184 and *XCR1*. Significantly, our study is the first to link non‐coding SNP with phase separation, which may provide new insight into the function and mechanisms of RUNX2 in osteoblast differentiation and bone, and may help accelerate the understanding of osteoporosis susceptibility. Our findings offer a novel perspective on the genetic mechanisms underlying osteoporosis by assessing the phase separation status of RUNX2. Moreover, our findings have the potential to enhance the accuracy of predicting therapeutic responses among individuals with different genotypes, thereby contributing to precision medicine for osteoporosis and advancing the development of personalized healthcare.

Non‐coding SNPs reside in *cis*‐regulatory elements and can act as enhancers to recruit TF to activate target genes expression.^[^ ^11^ ^]^ Interestingly, recent studies on phase separation of TFs have brought new enlightenment for the dynamic changes of 3D chromatin and transcriptional regulation.^[^ ^17^, ^18^ ^]^ 3D chromatin changes make the remote enhancer contact with the target promoter in cis through TFs to regulate gene expression, which is a fundamental mechanism underlying differential transcriptional regulation.^[^ ^13^, ^26^, ^27^ ^]^ LLPS can achieve specific aggregation of intracellular molecules, form independent microenvironments without a membrane, and improve the reaction rate of molecular interactions within condensates.^[^ ^18^, ^28^ ^]^ The assembly of dynamic membrane‐free compartments in LLPS plays a crucial role in gene expression regulation.^[^ ^20^ ^]^ It is worth noting that 1,6‐HD can inhibit the formation of phase separation condensates,^[^ ^16^, ^29^, ^30^ ^]^ decrease enhancer‐promoter interactions and topologically associating domain insulation, but not considerably affect CTCF‐dependent loops.^[^ ^29^ ^]^ 1,6‐HD‐sensitive LLPS have an effect on chromatin spatial organization by constraining its folding patterns and facilitating compartmentalization at different levels.^[^ ^29^ ^]^ In our study, when the phase‐separated droplet aggregates were disrupted by 1,6‐HD, the enhancing effect of SNP rs4683184 allele A on the activity of the *XCR1* promoter disappeared and resulted in a decrease in the expression of *XCR1*. In addition, we found the nascent *XCR1* RNA and SNP rs4683184 in the RUNX2‐IDR phase separation condensates. These findings implied that RUNX2‐IDR phase separation could be involved in the long‐range regulation of rs4683184 and target gene *XCR1*. Our study may provide new insights into the mechanisms of chromatin remote regulation and 3D genomic organization rearrangement.

As a vital member of the GPCR family, XCR1 can transmit signals by increasing intracellular calcium ion levels, and plays a critical role in dendritic‐cell‐mediated cytotoxic immune response, and in cancer cell proliferation and migration.^[^ ^31^, ^32^, ^33^ ^]^ However, the function and regulatory mechanism of *XCR1* in bone remain unclear. In osteoblasts, our study found that XCR1 not only promoted ALP activity and the formation of mineralized nodules, but also significantly promoted the expression of marker genes for osteoblast differentiation, suggesting that *XCR1* is a positive regulator of osteoblast differentiation, and might be a potential target for osteoporosis. In recent years, AAV has been widely used in gene therapy and vaccine research due to its low immunogenicity and long‐expression time of foreign genes in vivo.^[^ ^34^, ^35^ ^]^ By modifying the AAV capsid protein to obtain tissue‐specific serotype and make it recognize specific cell surface receptors, AAV‐specific infection of corresponding cells can be achieved, thus achieving specific gene regulation,^[^ ^34^, ^36^ ^]^ however, there are few researches on the bone targeting therapy of AAV.^[^ ^36^ ^]^ Here, we modified the AAV‐related vector, so that the modified AAV capsid protein can accurately recognize osteoblasts and achieve the purpose of targeted therapy. We used bone‐targeting AAV of *Xcr1* (AAV‐DSS‐Xcr1) to treat osteoporosis mice and found that *Xcr1* improved bone formation in osteoporosis mice. These findings suggest that *XCR1* may be a new drug target of osteoporosis, and also would provide a potential possibility for the targeted therapy of AAV in bone.

In summary, our study reveals that RUNX2 phase separation mediates the long‐range interaction between non‐coding susceptibility SNP rs4683184 and target gene *XCR1*, and then regulates the expression of *XCR1* involved in osteoblast differentiation and osteoporosis. These results not only help us to understand the remote transcriptional regulation process in osteoblast differentiation from a new perspective but also clarify a new mechanism by which transcription factors and non‐coding SNP affect disease susceptibility through phase separation. This strategy is expected to significantly accelerate the development of new drugs for osteoporosis, providing more effective treatment options for clinical practice.

## 4 Experimental Section

### GWAS Conditional Analysis and Cis‐eQTL Association Analysis

Quantitative heel ultrasounds eBMD related GWAS data were collected from the Genetic Factors for Osteoporosis Consortium (GEFOS) portal (http://www.gefos.org/?q=content/data‐release‐2018), and downloaded UK Biobank genotype data (Application number: 46387). Referring to the previous study,^[^ ^37^ ^]^ 50000 White British non‐relative samples were randomly chosen and filtered as genotype reference panels. Four conditionally independently associated SNPs (indSNP, adjusted *p* < 5 × 10^−8^) at 3p21.31 on eBMD (rs4683184, rs62261502, rs2624834, rs13072536) were collected from the publication of Morris et. al.^[^ ^38^ ^]^ The conditional association signal on rs4683184 was calculated by controlling for three other conditionally independent association signals using GCTA –cojo‐cond (v1.92.1).^[^ ^39^ ^]^ Genetic variants were annotated by ANNOVAR tool.^[^ ^40^ ^]^ Cis‐eQTL association in whole blood from publicly available Genotype‐Tissue Expression (GTEx) database (https://www.gtexportal.org/home/), corresponding genotype data obtained from dbGaP (phs000424.v8.p2). Cis‐eQTL analysis was then implemented between selected SNP and target gene.

### Motif Analysis

Motif analysis surrounding SNP was conducted by using MEME suite tool kit (University of California, San Diego, CA)^[^ ^41^ ^]^ with default parameters and TF motif from multiple databases. The motif with predicted allele‐specific binding at SNP rs4683184 was retained.

### Cell Line Culture, Primary Human Osteoblasts Culture

The human osteoblast‐like cell line MG‐63 and the 293T cells (HEK293T) were cultured in DMEM medium (HyClone, USA) supplemented with 10% fetal bovine serum (Biological Industries, Israel), Penicillin‐Streptomycin Solution (C0222, Beyotime, China) at 37 °C incubator with 5% CO_2_. Besides, primary human osteoblasts were isolated from human vertebra samples as previously described.^[^ ^42^ ^]^ Human vertebra samples were provided by Honghui Hospital, China. All procedures performed in this study involving human samples were performed under the ethical approval and patient permission, and were in accordance with the ethical standards of the Ethics Committee of Honghui Hospital. Primary human osteoblasts were cultured in αMEM medium (HyClone, USA) supplemented with 10% fetal bovine serum (Biological Industries, Israel) and Penicillin‐Streptomycin Solution (C0222, Beyotime, China) at 37 °C incubator with 5% CO_2_. For osteoblast differentiation induction, MG‐63 cells were induced 14 days by treatment with 50 µg mL^−1^ ascorbic acid (1043003, Sigma–Aldrich) and 10 mm β‐glycerophosphate (G9422, Sigma–Aldrich).

### Genotyping of SNP rs4683184 in Osteoblasts

The genomic DNA was isolated from MG‐63 cells, hFOB1.19 cells, and primary human osteoblasts by using TIANamp genomic DNA extraction kit (DP304, TIANGEN Biotech, China). The DNA fragments surrounding rs4683184 were amplified by PCR, and purified the DNA for Sanger sequencing. SNP rs4683184 was found to be homozygous AA in osteoblast‐like MG‐63 cell line, was homozygous GG in hFOB1.19 cells, and was heterozygous A/G in primary human osteoblasts. Subsequent cell experiments were performed in MG‐63 cells or primary human osteoblasts.

### Knockdown and Overexpression for Genes

Short hairpin RNA (shRNA) was used to knockdown the expression of *XCR1* and *RUNX2* in MG‐63 cells. Targeted sequences of shRNA are shown in Table S3 (Supporting Information). Lentivirus of shRNA was generated by HEK293T cells. The IDR sequence of RUNX2 was analyzed in PLAAC (http://plaac.wi.mit.edu/)^[^ ^43^ ^]^ and PONDER (http://pondr.com/).^[^ ^44^, ^45^ ^]^ Sequences of human XCR1 CDS, RUNX2 CDS, or IDR were cloned into vector to generate the recombinant plasmids. These recombinant plasmids and two helper plasmids (pCMV‐VSV‐G, cat no.8454 and 12260 cat no. 80054, Addgene, USA) were co‐transfected into HEK293T cells. Lentivirus of different group infected MG‐63 cells to regulate XCR1, RUNX2, and RUNX2‐IDR expression, individually. All target or primer sequences for gene knocking down and overexpression were listed in Table S3 (Supporting Information).

### RNA Isolation, and Real‐Time qPCR (RT‐qPCR)

Total RNA was isolated from the cells using fast 200 RNA isolation kit (220011, Fastgen, China) and was reverse‐transcribed into cDNA by using the ABScript III RT Master Mix for qPCR with gDNA Remover (RK20429, ABclonal, China). The qPCR reaction was performed by using the 2 × SYBR Green qPCR Kit (RK21203, ABclonal, China) and Bio‐Rad CFX Connect Real‐Time PCR Detection System (Bio‐Rad, USA). Glyceraldehyde 3‐phosphate dehydrogenase (*GAPDH*) was used as an endogenous control to normalize the different samples. All primers of RT‐qPCR are shown in Table S4 (Supporting Information).

### DNA Fragment Deletion by CRISPR‐Cas9

To efficiently delete the DNA fragments residing SNP rs4683184, CRISPR‐associated RNA‐guided endonuclease Cas9 cleavage technology was used in the study as described previously.^[^ ^46^ ^]^ CRISPR design platform maintained by the Zhang Lab at the Broad Institute (https://zlab.squarespace.com/guide‐design‐resources) was used. Different sgRNAs were cloned into lentiCRISPR v2 plasmid (52961, Addgene, USA). Different sgRNA group lentivirus and 8 µg mL^−1^ polybrene (H8761, Solarbio, China) were used to infect MG‐63 cells. After selection with puromycin (2 mg mL^−1^, ST551, Beyotime, China), the cells were used for further experiments. All primers of DNA fragment deletion are listed in Table S5 (Supporting Information). The CRISPR deletion efficiency was validated by gel electrophoresis experiments, sequencing, and TIDE analysis (https://tide.nki.nl/).

### Alkaline Phosphatase (ALP) and Alizarin Red S (ARS) Staining

Cells were fixed with 4% paraformaldehyde solution for 20 min. ALP staining was performed with BCIP/NBT alkaline phosphatase color development kit (C3206, Beyotime, China) according to the manufacturer's instructions. For ARS staining, fixed cells were incubated with 2% Alizarin Red S Staining Solution (PH 4.2, C0138, Beyotime, China) according to manufacturer's instructions.

### Western Blot

Proteins from MG‐63 cells were loaded on SDS‐PAGE and then electro‐transferred on polyvinylidene fluoride membranes. Primary antibodies were used as below: anti‐XCR1 (1:1000, YN0292, ImmunoWay Biotechnology, USA), anti‐SP7 (1:400, sc‐393325, Santa Cruz, USA), anti‐RUNX2 (1:500, 20700‐1‐AP, Proteintech, China) and anti‐GAPDH (1:1000, ET1601‐4, Huabio, China). Secondary antibodies were HRP‐labeled goat anti‐rabbit IgG(H+L) (1:5000, LF102, EpiZyme, China) and HRP‐labeled goat anti‐mouse IgG(H+L) (1:5000, LF101, EpiZyme, China). Protein marker (WJ103, EpiZyme, China) was purchased from EpiZyme Company. Fast stripping buffer (PS107, EpiZyme, China) was used according to the manufacturer's instructions. Protein band intensities were detected by quantity one software (Bio‐Rad, USA), and evaluated by ImageJ software (USA). Related original images are shown in Figure S5 (Supporting Information).

### Dual‐Luciferase Reporter Assay

*XCR1* promoter fragments were inserted into pGL3‐basic vector. Further, DNA sequences containing SNP rs4683184 were cloned and inserted into the upstream of the pGL3‐XCR1‐promoter vector. Besides, in order to construct the major allele type and minor allele type vector, respectively, site‐directed mutagenesis was performed with the quick change II site‐directed mutagenesis Kit (200523, Agilent Technologies, USA) according to the manufacturer's instructions. Related primers are shown in Table S6 (Supporting Information). Related constructed plasmids are shown in Figure S2E (Supporting Information). Sequencing was further performed to verify the recombinant plasmids with correct sequences. The constructed expression plasmids and pRL‐TK (internal control) were transfected into MG‐63 cells and primary human osteoblasts by using Lipo8000 transfection reagent (C0533, Beyotime, China) according to the manufacturer's instructions. And then, the transfected cells were incubated in 5% CO_2_ at 37 °C incubator. After 48 h of transfection, Firefly and renilla luciferases were detected using the Dual‐Luciferase Reporter Assay System (RG028, Beyotime, China).

### Chromosome Conformation Capture (3C)‐qPCR and Chromatin Immunoprecipitation (ChIP) ‐qPCR Assay

The chromosome conformation capture (3C) was performed as described previously^[^ ^47^ ^]^ in MG‐63 cell lines. Briefly, MG‐63 cells were fixed with formaldehyde and 2.5 m glycine was added to stop the crosslinking. Cells were collected and centrifuged, and the supernatant was removed. 1 × rCutSmart buffer (B6004S, NEB, USA) was added to the samples and digested using Pst I‐HF restrictive enzymes (20000 units mL^−1^, R3140S, NEB, USA). After that, the restriction enzyme was inactivated and the cooled ligation cocktail was used in our samples. T4 DNA ligases (400 000 units mL^−1^, M0202S, NEB, USA) were added to our digestive products, and then the DNA was purified. Finally, a control library was prepared, and the frequency of DNA interaction was detected by qPCR. Besides, ChIP assay was performed using the SimpleChIP Enzymatic Chromatin IP Kit (9003S, Cell Signaling Technology, USA) in primary human osteoblasts and MG‐63 cells as described by manufacturer's instructions. For immunoprecipitation, anti‐RUNX2 (20700‐1‐AP, Proteintech, China) was used. Related primer sequences are presented in Table S7 (Supporting Information).

### Cell Immunofluorescence (IF) Staining

MG‐63 cells were seeded on coverslips and cultured for 24 h. The cells were treated with 1.5% 1,6 HD (240117, Sigma–Aldrich, USA) at different times. After 15 min fixation with 4% PFA and permeated with 0.5% Triton X‐100 (P1080, Solarbio, China), cells were blocked with 1% bovine serum albumin (BSA) (ST2254, Beyotime, China) in PBS for 30 min at room temperature, then incubated with anti‐XCR1 (1:200, YN0292, ImmunoWay Biotechnology, USA) or anti‐RUNX2 (1:150, 20700‐1‐AP, Proteintech, China) overnight at 4 °C. Secondary antibodies were goat anti‐rabbit IgG(H+L)‐FITC (1:200, RS0004, ImmunoWay Biotechnology, USA) or goat anti‐mouse IgG(H+L)‐FITC (1:200, RS0003, ImmunoWay Biotechnology, USA). Actin‐Tracker Red‐Rhodamine (C2207S, Beyotime, China) was used in accordance with the manufacturer's instructions. DAPI (C1002, Beyotime, China) was used to counterstain cell nuclei for 10 min at room temperature. Then, samples were examined with the Leica TCS SP8 STED 3X laser scanning confocal microscope (Leica, Germany). IgG was used as the control for the IF experiment instead of the primary antibody, related images are shown in Figures S1A and S4A (Supporting Information). Fluorescence quantification analysis was performed by ImageJ software.

### 3D‐DNA FISH, IF‐DNA FISH and IF‐RNA FISH Experiment

The DNA‐FISH probes for DNA fragments containing SNP rs4683184 or *XCR1* promoter were designed using PaintSHOP,^[^ ^48^ ^]^ respectively. DNA‐FISH probes are listed in the Table S8 (Supporting Information). Further, 3D‐DNA FISH and IF‐3D‐DNA FISH were performed in M6‐63 cells as previously described.^[^ ^49^ ^]^ For RNA‐FISH, intron RNA probes of *XCR1* were used and labeled with Digoxin (FOCOFISH, Guangzhou, China), and the sequences were listed in Table S9 (Supporting Information). Besides, TSA‐488 Fluorescence System Kit (D‐0012F) and TSA‐555 Fluorescence System Kit (D‐0012R) were purchased from FOCOFISH company (Guangzhou, China). Images were captured by the Leica TCS SP8 STED 3X laser scanning confocal microscope (Leica, Germany) and the Meagle 100 professional edition laser scanning confocal microscope (Changyi Guangke, China). IgG was used as the control for the IF experiment instead of the primary antibody, the group without a probe as the control for the DNA‐FISH experiment, and the U6 probe as the control for the RNA‐FISH experiment. Related controls of FISH experiments were also shown in Figure S4B–D (Supporting Information). Fluorescence colocalization analysis was performed by ImageJ software.

### Generation of Bone‐Targeting rAAV9 Vector and Animal Experiment

The sequences of bone‐targeting peptide motif DSS (AspSerSer)_6_ were inserted into AAV capsid proteins, bone‐targeting *Xcr1* overexpression vector was constructed and generation of rAAV‐DSS‐Xcr1 virus as described by Yeon‐Suk Yang et al.^[^ ^36^ ^]^ 3 months old female C57BL/6J mice were executed OVX surgery or removed 1 g fat near the ovaries (sham treatment). After 30 days, 10 µl rAAV‐DSS‐Xcr1 or rAAV‐DSS‐Gfp (titer > 10^11^ mL^−1^) were I.A. injected into knee joints of OVX mice. The workflow of OVX and subsequent rAAV treatment is shown in Figure 6A. Moreover, GFP expression in different tissues of rAAV‐DSS‐Gfp treatment mice was detected by IVIS Lumina XRMS Series III In Vivo Imaging System, in order to confirm the bone‐targeting efficiency of rAAV‐DSS. In the study, all experiments used wild type (WT) C57BL/6 female mice that were purchased from the Animal Care of Xi'an Jiaotong University. All animal experiments were performed according to the requirements and regulations of the Animal Care of Xi'an Jiaotong University.

### Micro‐Computed Tomography (Micro‐CT) Analysis

Femurs without muscle and connective tissue were isolated from mice, and cleaned femurs were fixed in 4% paraformaldehyde solution and stored at 4 °C. Then, distal femurs were scanned with Quantum GX2 micro‐CT Imaging System (PerkinElmer, USA), and parameters are as follows: X‐ray kV (90 kV), X‐ray µA (88 µA), FOV (7.68 mm) and pixel size is 15 µm.

### Immunohistochemistry (IHC) Staining

Paraffin bone sections were deparaffined and were further carried out IHC staining. Besides, antigens retrieval of slides was achieved by heat treatment with a commercial reagent (P0083, Beyotime, China). Primary antibodies used for the staining were anti‐XCR1 (1:150, YN0292, ImmunoWay Biotechnology, USA), anti‐SP7 (1:150, sc‐393325, Santa Cruz, USA), anti‐RUNX2 (1:150, 20700‐1‐AP, Proteintech, China), anti‐COL1α1 (1:150, PAB46098, Bioswamp, China). The average optical density (AOD) is ratio of the integral optical density (IOD) to the area. AOD analysis of IHC staining was performed using ImageJ software.

### Statistical Analysis

Experimental data were reported as averages ± SD by using GraphPad Prism 8.0 (GraphPad, San Diego, California, USA). Data were analyzed with two‐tailed unpaired *t*‐test. *p* values <0.05 were considered significant in the study. *, *p* < 0.05; **, *p* < 0.01; ***, *p* < 0.001.

### Ethics Approval Statement

All animal experiments involving the use of animals were approved by the Animal Care of Xi'an Jiaotong University, ethical approval number: XJTUAE2023‐664. Primary human osteoblasts were isolated from human vertebra samples, all procedures performed in this study were in accordance with the ethical standards of the Ethics Committee of Honghui Hospital, and ethical approval number: 202304005.

## Conflict of Interest

The authors declare no conflict of interest.

## Author Contributions

Y.Z., XH.L., and P.P. contributed equally to this study. Y.G., TL.Y., and Y.Z. designed the study. Y.Z., XH.L., P.P., ZH.Q., CX.D., NN.W., and F.C. collected data and performed analysis. Y.Z., F.Z., YW.H., ZB.L., DL.Z., SY.H., Z.Y., and YP.L. gave experimental technical support. X.F.C. and S.S.D. performed bioinformatic analysis. Y.Z., Y.G., and TL.Y. drafted the manuscript. All authors approved the final version of the manuscript for submission.

## Supporting information



Supporting Information

## Data Availability

The data that support the findings of this study are available from the corresponding author upon reasonable request.
