# Supplementary material for: RUNX2 Phase Separation Mediates Long‐Range Regulation Between Osteoporosis‐Susceptibility Variant and XCR1 to Promote Osteoblast Differentiation
Source: Adv Sci (Weinh). 2024 Dec 20;12(6):2413561. doi: 10.1002/advs.202413561 (PMC11809430; doi:10.1002/advs.202413561)
Supplement: Supplementary file 1 — Supporting Information [file ADVS-12-2413561-s001.docx]

**Title：RUNX2 Phase Separation Mediates Long-Range Regulation between Osteoporosis-Susceptibility Variant and *XCR1* to Promote Osteoblast Differentiation**

Yan Zhang, Xin-Hao Li, Pai Peng, Zi-Han Qiu, Chen-Xi Di, Xiao-Feng Chen, Nai-Ning Wang, Fei Chen, Yin-Wei He, Zhong-Bo Liu, Fan Zhao, Dong-Li Zhu, Shan-Shan Dong, Shou-Ye Hu, Zhi Yang, Yi-Ping Li, Yan Guo, Tie-Lin Yang

**This PDF file includes:**

Experimental Section

Supplementary Figures 1-5

Supplementary Tables 1-9

**Experimental Section**

**Functional epigenetic annotation**

To study potential functional variants, several active enhancer-related epigenetic markers were annotated for the regions surrounding SNP rs4683184 using ChIP sequencing data from ENCODE (ENCODE_Project_Consortium, 2012), including the histone markers and histone acetyltransferase p300 binding sites in osteoblasts and GM12878 respectively. All annotated data were visualized by using WashU EpiGenome Browser (http://epigenomegateway.wustl.edu/browser/) (version 46.2; Washington University, St.Louis, MO).

**Cell proliferation assay by using Cell-Counting Kit-8 (CCK-8)**

The different group cells were seeded in 96-well plates. CCK-8 solutions (40203ES60, Yeasen, China) were added to each well at different time point, and incubated 1 h at 37 °C. Then, we detected the optical density (OD) value at 450 nm by using a microplate reader (MULTISKAN FC, Thermo scientific, USA).

**Supplemental figures**

**
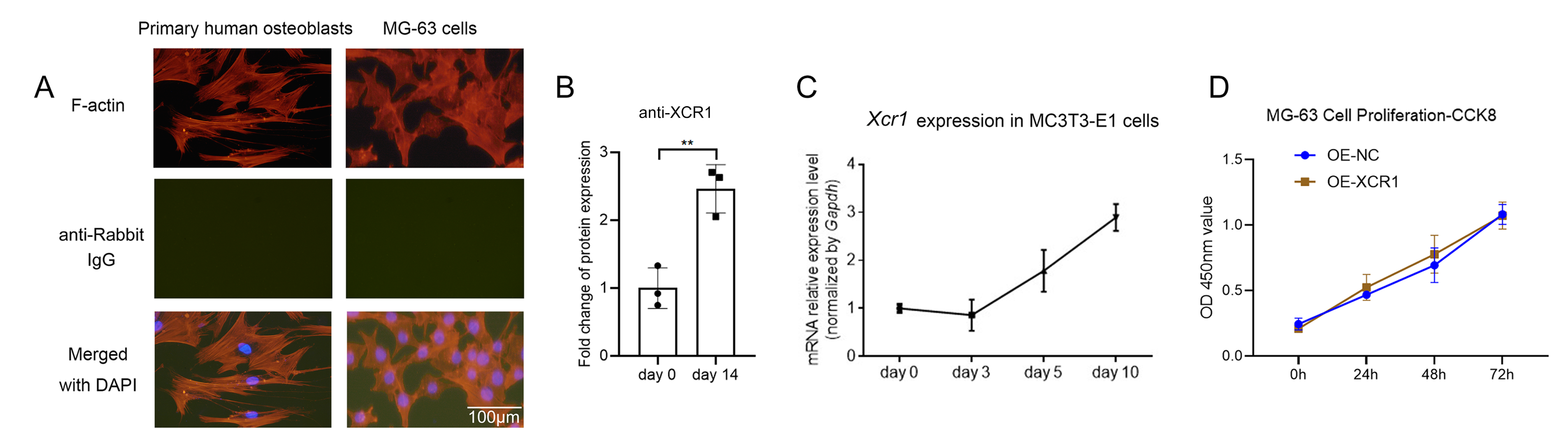
**

**Figure S1.** XCR1 affected osteoblast differentiation rather than osteoblast proliferation. A) We used IgG as the negative control for the Figure 1C instead of the primary antibody. Bar: 100 µm. B) Fold change of XCR1 protein expression for the Figure 1D in MG-63 cells were induced osteoblast differentiation for 14 days. C) RT-qPCR detected the *XCR1* mRNA expression in mouse osteoblastic line MC3T3-E1 at different induction days. D) CCK8 experiment showed *XCR1* has no significant effect on MG-63 cells proliferation. ^**^*P* < 0.01.

**
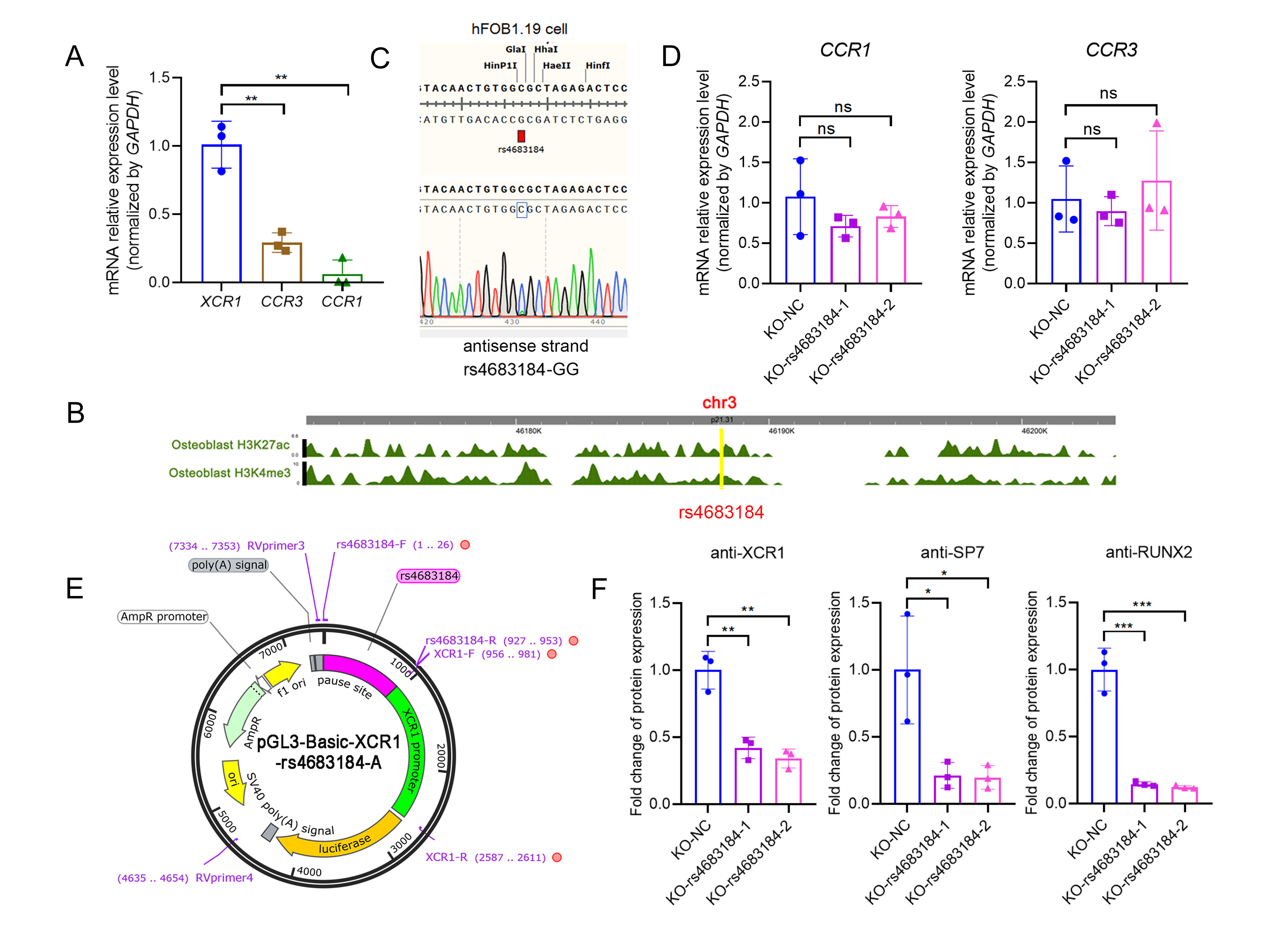
**

**Figure S2.** *XCR1* could be the target gene for SNP rs4683184 in osteoblasts. A) RT-qPCR detected the *XCR1*, *CCR1* and *CCR3* mRNA expression in human osteoblastic line MG-63. B) Epigenetic annotation for the region surrounding SNP rs4683184 in osteoblasts, visualized by the WashU Epigenome Browser. C) The genotype of rs4683184 in human osteoblast-like hFOB1.19 cell line. D) mRNA expression levels of *CCR1* and *CCR3* were detected by RT-qPCR in control (KO-NC) and in rs4683184 knockout (KO-rs4683184) MG-63 cells. E) The plasmid construction diagram of pGL3-XCR1-promoter-rs4683184-A for dual-luciferase reporter assays. F) Fold change of XCR1, SP7 and RUNX2 protein expression in control (KO-NC) and in rs4683184 knockout (KO-rs4683184) MG-63 cells. Values of *P* were determined with a two-tailed t-test. ns. no significant difference. ^*^*P*< 0.05, ^**^*P* < 0.01, ^***^*P*< 0.001.

**
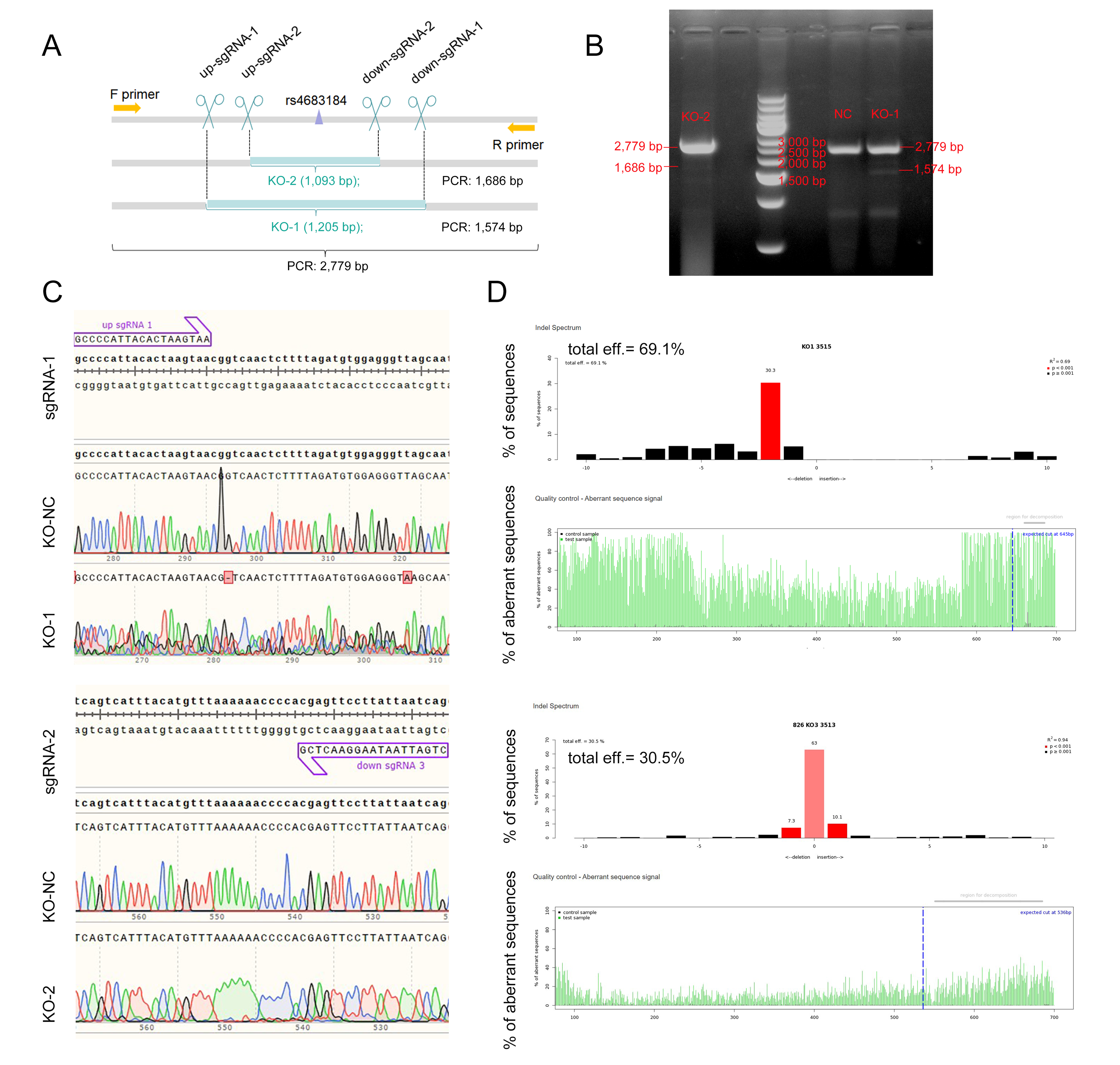
**

**Figure S3.** Knockout efficiency of the region containing SNP rs4683184. A) The scheme for deleting the region containing rs4683184 by CRISPR/Cas9. Two pairs of sgRNAs were designed. The original PCR amplified fragment was 2,779 bp, when some fragments were knocked out, a 1,574 bp (sgRNA1) or 1,686 bp (sgRNA2) fragment would be generated. B) The gel result of PCR showed the knockout efficiency of rs4683184. C,D) Knockout efficiency of the region containing rs4683184 by sequencing and TIDE analysis.

**
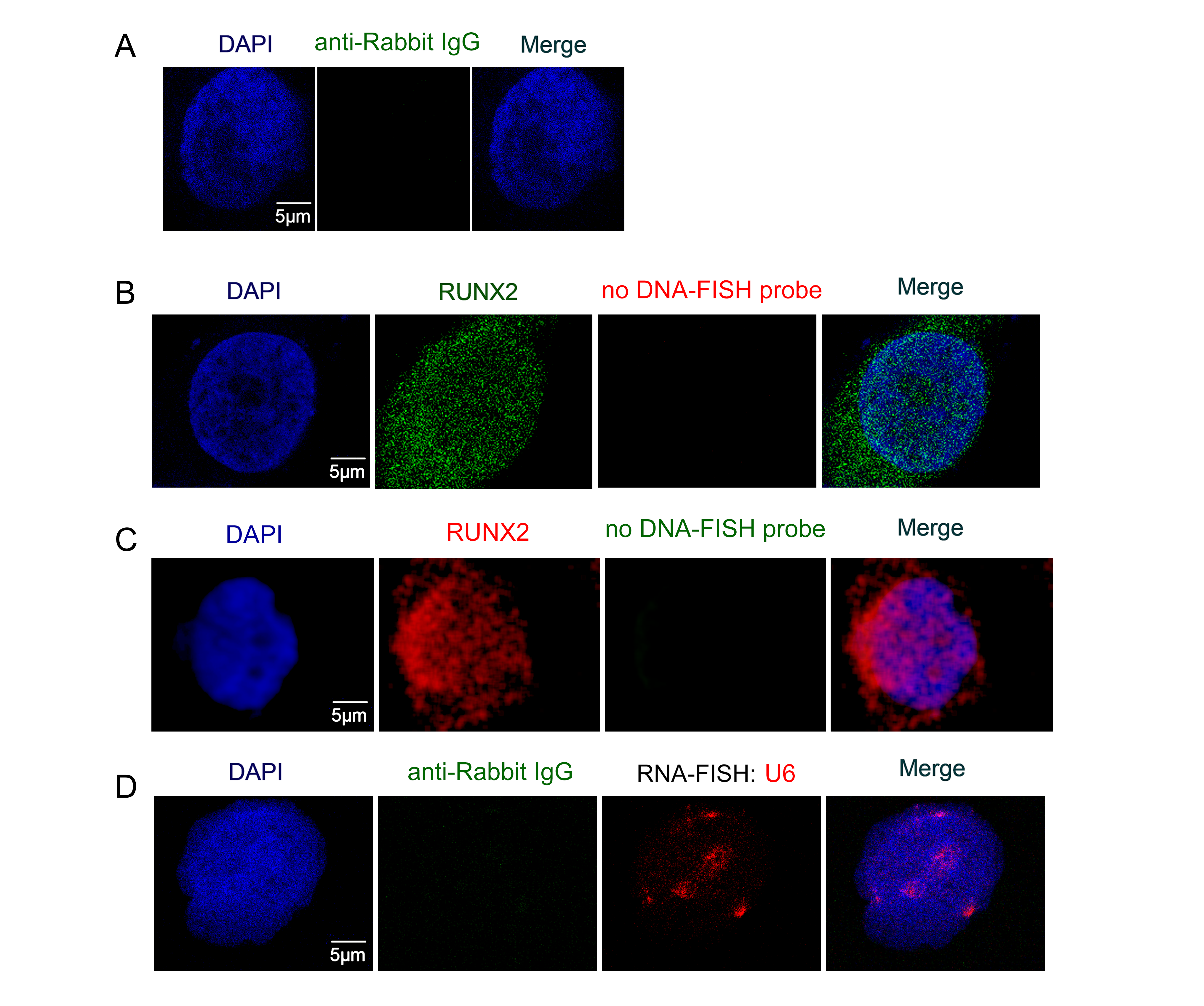
**

**Figure S4.** Controls of immunofluorescence (IF) staining, DNA-FISH and RNA-FISH experiments in MG-63 cells. A) IgG was used as the control for the IF experiments shown in Figures 4D and 5C. Bar: 5 µm. B) The group without a DNA probe served as the control for the DNA-FISH experiment shown in Figures 4E and 5F. Green: anti-RUNX2. Bar: 5 µm. C) The group without a DNA probe served as the control for the DNA-FISH experiment shown in Figures 4F and 5G**.** Red: anti-RUNX2. Bar: 5 µm. D) U6 probes were used as the control for the RNA-FISH experiment shown in Figures 4G and 5H**.** Red: U6 probes. Bar: 5 µm.

**
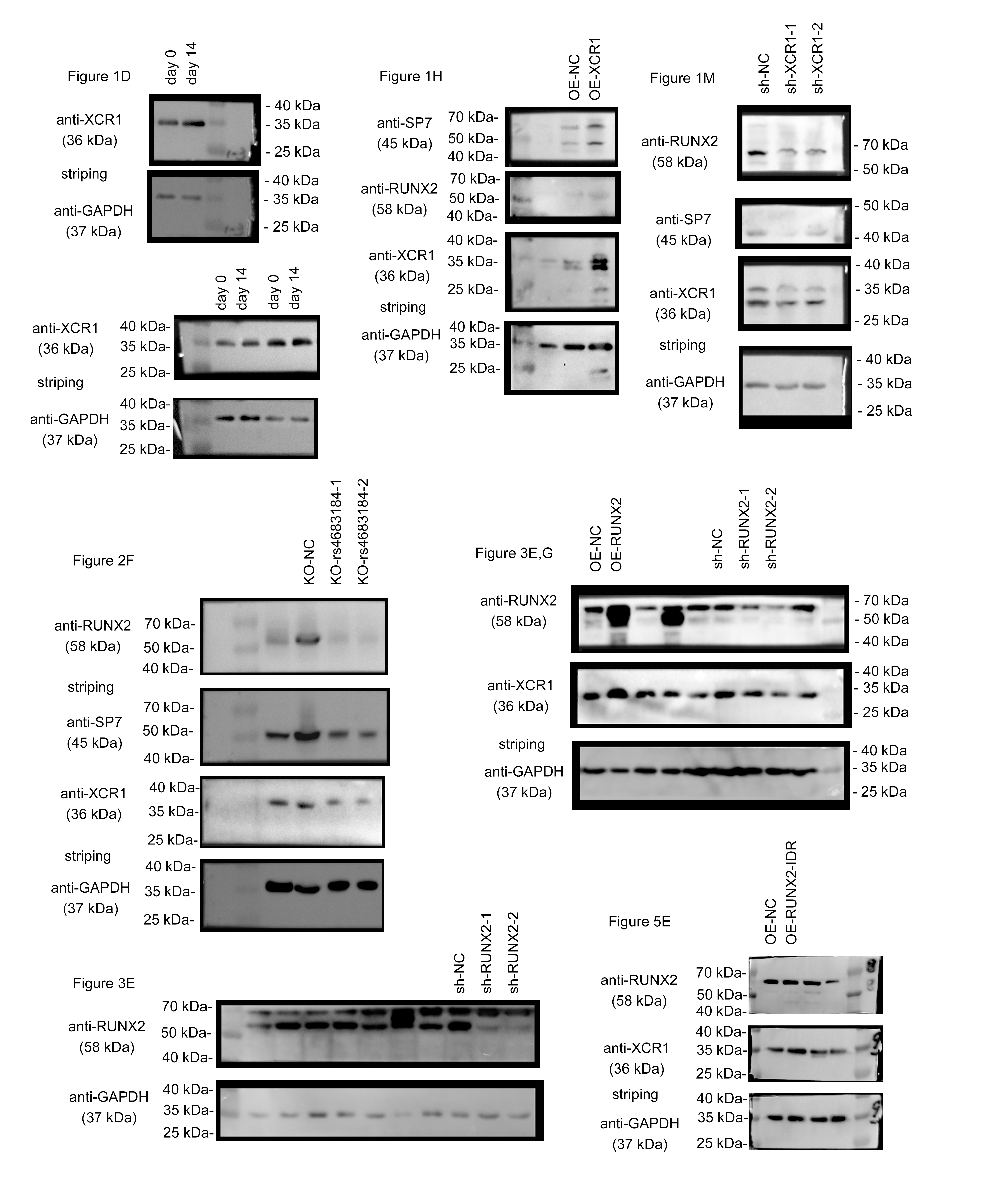
**

**Figure S5.** Original images of western blot.

**Supplemental Tables**

**Table S1** The eQTL analyses of SNP rs4683184 on gene in whole blood

| **Gene** | **SNP** | **Effect.allele** | **other.allele** | **β** | **SE** | **P value** |
| --- | --- | --- | --- | --- | --- | --- |
| ENSG00000163823_CCR1 | rs4683184 | A | G | -0.34976 | 0.0231569 | 6.41×10^-44^ |
| ENSG00000183625_CCR3 | rs4683184 | A | G | 0.379483 | 0.0321468 | 4.60×10^-29^ |
| ENSG00000173578_XCR1 | rs4683184 | A | G | 0.224564 | 0.0379675 | 5.59×10^-9^ |

*Notes*: β: β coefficient of eQTL, SE: standard error of β coefficient.

**Table S2** The eQTL analyses of 9 SNPs (in Table1) on *XCR1* in whole blood

| **Gene** | **SNP** | **β** | **SE** | **P value** |
| --- | --- | --- | --- | --- |
| ENSG00000173578_XCR1 | rs4683184 | 0.224564 | 0.0379675 | 5.59×10^-09^ |
| ENSG00000173578_XCR1 | rs2172247 | 0.230227 | 0.038492 | 3.81×10^-09^ |
| ENSG00000173578_XCR1 | rs7616215 | 0.230227 | 0.038492 | 3.81×10^-09^ |
| ENSG00000173578_XCR1 | rs2087726 | 0.230227 | 0.038492 | 3.81×10^-09^ |
| ENSG00000173578_XCR1 | rs2201150 | 0.242467 | 0.0376871 | 2.55×10^-10^ |
| ENSG00000173578_XCR1 | rs7648466 | 0.207685 | 0.0416313 | 7.97×10^-07^ |
| ENSG00000173578_XCR1 | rs2172246 | 0.209107 | 0.0419841 | 8.30×10^-07^ |
| ENSG00000173578_XCR1 | rs1491961 | 0.211459 | 0.0422429 | 7.32×10^-07^ |
| ENSG00000173578_XCR1 | rs3181077 | 0.215736 | 0.0421118 | 4.06×10^-07^ |

*Notes*: β: β coefficient of eQTL, SE: standard error of β coefficient.

| **Table S3** shRNA target sequences and primers for gene overexpression (OE) | |
| --- | --- |
| **Name** | **Target or primer sequence (5'-3')** |
| sh-NC | CGCTGAGTACTTCGTAATGTC |
| sh-XCR1-1 | CCTCAATATGATCTTCTCCAT |
| sh-XCR1-2 | CCCTACAACTTCACCCTGTTT |
| sh-RUNX2-1 | CAGCACTCCATATCTCTACTA |
| sh-RUNX2-2 | GTGGTCCTATGACCAGTCTTA |
| OE-XCR1-CDS-F-EcoRI | CCGCAATTCATGGAGTCCTCAGGCAACC |
| OE-XCR1-CDS-R-XhoI | CCGCTCGAGTCAGTAGAAGGAGGCGCC |
| OE-RUNX2-CDS-F-XbaI | GCTCTAGAGCCACCATGGCATCAAACAGCCTCTTCAGC |
| OE-RUNX2-CDS-R-XhoI | CCCTCGAGTCAATATGGTCGCCAAACAGATTC |
| OE-RUNX2-IDR-F-XbaI | GCTCTAGAGCCACCATGGCATCAAACAGCCTCTTCAGC |
| OE-RUNX2-IDR-R-XhoI | CCCTCGAGTCACTCCACCATGGTGCGGTTGTCGTG |

| **Table S4** RT-qPCR primer sequences | |
| --- | --- |
| **Primer name** | **Primer sequence (5'-3')** |
| human-XCR1-qPCR-F | ATGGAGTCCTCAGGCAACC |
| human-XCR1-qPCR-R | CGAGGGTAGCAAAGACCCA |
| human-GAPDH-F | GGAGCGAGATCCCTCCAAAAT |
| human-GAPDH-R | GGCTGTTGTCATACTTCTCATGG |
| human-SP7(OSX)-F | CCTCTGCGGGACTCAACAAC |
| human-SP7(OSX)-R | AGCCCATTAGTGCTTGTAAAGG |
| human-ALP(ALPL)-F | GCTGTAAGGACATCGCCTACCA |
| human-ALP(ALPL)-R | CCTGGCTTTCTCGTCACTCTCA |
| human-RUNX2-F | GCGCATTCCTCATCCCAGTA |
| human-RUNX2-R | GGCTCAGGTAGGAGGGGTAA |
| human-COL1α1-F | TTTGGATGGTGCCAAGGGAG |
| human-COL1α1-R | AGTAGCACCATCATTTCCACGA |
| human-OCN-F | AGCGAGGTAGTGAAGAGAC |
| human-OCN-R | GAAAGCCGATGTGGTCAG |
| human-CTNNB1-F | CCCACTAATGTCCAGCGTTT |
| human-CTNNB1-R | AACGCATGATAGCGTGTCTG |
| human-CCR1-F | GACTATGACACGACCACAGAGT |
| human-CCR1-R | CCAACCAGGCCAATGACAAATA |
| human-CCR3-F | TGGCATGTGTAAGCTCCTCTC |
| human-CCR3-R | CCTGTCGATTGTCAGCAGGATTA |

| **Table S5** sgRNA sequences and PCR primers for rs4683184 knockout (KO) | |
| --- | --- |
| **Name** | **Sequence (5'-3')** |
| sgRNA-NC-up | ACCGATCGACTAGCCACTCAGACA |
| sgRNA-NC-down | TAACTGTCTGAGTGGCTAGTCGAT |
| rs4683184-KO-up-sgRNA-1 | GCCCCATTACACTAAGTAA |
| rs4683184-KO-down-sgRNA-1 | GTACTCCTTATATAGCCGT |
| rs4683184-KO-up-sgRNA-2 | GTACTGACTCCTAATAGAT |
| rs4683184-KO-down-sgRNA-2 | CGAGTTCCTTATTAATCAG |
| rs4683184-KO-PCR-F | GCCAGGGAACGTTGCACTAC |
| rs4683184-KO-PCR-R | CACGCATGCATATCCAACCC |

| **Table S6** Primers for dul-luciferase reporter gene assay | |
| --- | --- |
| **Primer name** | **Primer sequence (5'-3')** |
| rs4683184-F | GGGGTACCAAGGAACTCGTGGGGTTTTT |
| rs4683184-F | CGAGCTCGTTGTGGAGGCCAGAAGTTTG |
| XCR1 promoter-F | CGACGCGTCCATGATCGCACCACTACAC |
| XCR1 promoter-R | CCGCTCGAGGGTGGAACTCCTTCAAACC |
| rs4683184-(A→G)-F | TCTCTAGCgCCACAGTTGTACACTACAGTATCTTTGG |
| rs4683184-(A→G)-R | AACTGTGGcGCTAGAGACTCCTGCCACCCTCA |
|  | |

| **Table S7** 3C experiment and ChIP-qPCR primer sequences | | |
| --- | --- | --- |
| **Experiments** | **Primer name** | **Primer sequence (5'-3')** |
| 3C amplification primers for control library | N1-F | TTCAACAACCGGAAACCTGC |
|  | N1-R | GCAACACAATAGTCGTAGGGG |
|  | N2-F | AGGGTTCATCTGACCTAGCA |
|  | N2-R | CCAGACCCTGTTTGTTTAGTCC |
|  | N3-outer-F | TGGGAAGAAGGAGTGTCCCT |
|  | N3-F | TTCAGCAGCCCCATTACACT |
|  | N3-R | TGGTACCAGTACCATGCTGTTTTG |
|  | N4-F | CCATATTCCTTTCCCCTCGC |
|  | N4-R | TGATAGCCAGTTTTATGTGCCA |
|  | N5-outer-F | GAAGACATATGTAAATAGAAGGACTTTCAGTGTTC |
|  | N5-F | ATCAGTTGACAATCCCAGCACT |
|  | N5-R | CCCAACTGACTTCTAGACTCTGA |
|  | N6-F | GGCGATAATGATGACGGTGC |
|  | N6-R | ACTTCCTAAACACACTGCGC |
|  | N7-F | ATTCGATGAGGCAGAGCCAC |
|  | N7-R | AACCACGGTGCTAAACTGCT |
| 3C-qPCR primers | N3-SNP-F | GTTTGGCAACTGTTGAGCGAGATGT |
|  | N3-SNP-R | GCAGAGTCTTTTAGTCTCCAAAGG |
|  | N1-R | GGGCTATGTTTCAAGTCACACA |
|  | N2-R | CTGCCAAACACTTTTAAACCCA |
|  | N4-R | CCCTATCAGAGACTCAGTGTTG |
|  | N5-XCR1 promoter-R | AATGAAATACAATGGGGAAAGCC |
|  | N6-R | CTGGGCCCATCCAGGCTTAACAAA |
|  | N7-R | AAACTGAACCTCAGTCAGCTCATAG |
| ChIP-RUNX2 and rs4683184-qPCR primers | No binding-NC-F | TGATGTCCATTGTCCTCTCCT |
|  | No binding-NC-R | TGGTGGATTCTTTCAGTTCTCT |
|  | Binding-F | ATAGCCGTAGGCACCACTTCT |
|  | Binding-R | GTCTCTAGCGCCACAGTTGTA |

| **Table S8** 3D-DNA-FISH probes | |
| --- | --- |
| **Probes name** | **Sequence (5'-3')** |
| XCR1 promoter-DNA-1 | CCTGAATCTACACCTCTTTACATGCGTCTTGA |
| XCR1 promoter-DNA-2 | TCACTTAAAGAAACTGAAACACCTGTCCTGCC |
| XCR1 promoter-DNA-3 | ACTGTTCCCCAATACTCCCACTCACTTCCATG |
| XCR1 promoter-DNA-4 | CTCAACAATCATGCATAATGCCGGCACCCC |
| XCR1 promoter-DNA-5 | CAACCCCATACATTCAGTCCAAACCTCCCTCT |
| XCR1 promoter-DNA-6 | GCTACAGATCTACAGTGGTCAACCTTGGTGTA |
| XCR1 promoter-DNA-7 | TAGCACCACCCCAGACTCAACTAACACCAAAC |
| XCR1 promoter-DNA-8 | CACATGCTGGCCTGGAGCATGCAATGTCTT |
| XCR1 promoter-DNA-9 | GTCATGTTGCTCCTGCCTGAAGTCCCCAGT |
| XCR1 promoter-DNA-10 | ATCTGCCTCCTACTTCCAGTGCCCACACTT |
| XCR1 promoter-DNA-11 | TAGGTGAGTCTGGCTTATGTTACTTAGGCATG |
| XCR1 promoter-DNA-12 | GCATGCATGTAGAGGGATGGGAAGAACTATTG |
| rs4683184-DNA-1 | CCCCACGGTGCAGCTGGACTTCCTTCTCAA |
| rs4683184-DNA-2 | GGAGAGGAGGCCTTCAGGGCACCACATGGG |
| rs4683184-DNA-3 | GAAGATAAGGGGTTGTGTGCCCAGCCCGGG |
| rs4683184-DNA-4 | GGGAAGGCTGCAGGATGGAGTAGGAAGCAAA |
| rs4683184-DNA-5 | CAAAGCAGTGGAGGGAGGTAGGGCTGCCCG |
| rs4683184-DNA-6 | GCAGGGACAGTGAAAGCTCAATGTGGACCAAATC |
| rs4683184-DNA-7 | ACAGCATTCTTTGGAAAGGAGTCTGTGTCCTTTCAGA |
| rs4683184-DNA-8 | ACCTTTTGAGATGTCCTGTAACCACTTCCACAGAGTT |
| rs4683184-DNA-9 | GAGTGGAGACCAGGGACAGCTGATGCCAAG |
| rs4683184-DNA-10 | TGAGAAAGAACAAAAGGAGACCTAGGAGGGCAGAACT |
| rs4683184-DNA-11 | TCATGGATGTGTACATGGGTGTGCAAAAGGGAAAA |
| rs4683184-DNA-12 | AGCACTGCAGGCAGTAGTGGGCACAGAGGT |
| rs4683184-DNA-13 | CAAAGTAGTGCAACGTTCCCTGGCCTGGGC |
| rs4683184-DNA-14 | GCTTCCCTTCTTTTGTGGAAGCCAAAGATCAATACCA |
| rs4683184-DNA-15 | CCTGAACTGATATCCATCAACAGCGCTTACCATCTCA |
| rs4683184-DNA-16 | CCAAGTGCTGGGTAGATGGTAGGAGAGTAGACTACAT |
| rs4683184-DNA-17 | AGTTGACCGTTACTTAGTGTAATGGGGCTGCTGAATT |
| rs4683184-DNA-18 | GCCCAAGACCTAAGAACTGAACAGCAGATACTGAAGA |
| rs4683184-DNA-19 | AGGGTGTCCTTATTCTGAGTCAGCCAACTTCTGAGTA |
| rs4683184-DNA-20 | ACCCCTTTGGAGACTAAAAGACTCTGCAGAATGTCAA |
| rs4683184-DNA-21 | GGCAACTGTTGAGCGAGATGTTACAAGACTAGAGTCA |
| rs4683184-DNA-22 | CAGATGCTTGATACCATGATGTCCTCACAGCATCTGA |
| rs4683184-DNA-23 | GTCACAATTTCCATCCTTCCTAGAGCTAGCCATCCAA |
| rs4683184-DNA-24 | CACAAGTGCTTCCAAGAATGAGCACCTTCTGCTTAAA |
| rs4683184-DNA-25 | ACTCCTGCCACCCTCAAGATGAAAGGCAAC |
| rs4683184-DNA-26 | CCCAAAGATACTGTAGTGTACAACTGTGGTGCTAGAG |
| rs4683184-DNA-27 | TGTCTGTAACTACACATGTACCCCTGCTGGCC |
| rs4683184-DNA-28 | CCCTGTACTCCTTATATAGCCGTAGGCACCACTTCTT |
| rs4683184-DNA-29 | CTGATGGTATCTGACTAGCAGCAGTGATGTGAGTTGT |
| rs4683184-DNA-30 | CCAGCCCTGGCTTCTCTCAATTGAAAGAAATGTAGGT |
| rs4683184-DNA-31 | TTTTGCGGCTGATTAATAAGGAACTCGTGGGGTTTT |
| rs4683184-DNA-32 | GGTGAACACCTTCTAGATGGTCTGATATGTGCTTGGT |
| rs4683184-DNA-33 | CCGGAACTCCCCAATACAGAACTCACTCTGCAAAA |
| rs4683184-DNA-34 | GCTTTTGCAGAGGGCAACAACTCTCTCTTACTTCCT |
| rs4683184-DNA-35 | TGGTAAAGCCCAAAATGCTAAGGGTACATGCGTATGA |
| rs4683184-DNA-36 | TGTCAACAGGAGCACACTTGTTCATGCCCAT |
| rs4683184-DNA-37 | ACCTCCCTGGAAGTGCCAGAAATCTTGTGAAATAAGA |
| rs4683184-DNA-38 | AAGCCCAGGCCTGATGAATGGACACCTGCA |
| rs4683184-DNA-39 | CTACTGCTCTGCTGGTTGTGTCGGGGAACA |
| rs4683184-DNA-40 | GGCAGCTCTGAGAGCTGTTGTGTCTGGGGA |
| rs4683184-DNA-41 | CTCATGCCAGTGGACAAATCAGGGCTTTTCGTA |
| rs4683184-DNA-42 | ACGCATGCATATCCAACCCTCCTTCCCTCA |
| rs4683184-DNA-43 | TCACCGCAGACATTGCTGAATGACTAAACCGAC |
| rs4683184-DNA-44 | AAGGTGTCAAGGACGCCAGCAGGCAGCTGTC |

| **Table S9** RNA-FISH probes | |
| --- | --- |
| **Probes name** | **Sequence (5'-3')** |
| human-XCR1-1 | AGAAGAGGTTGTGCTGGTAGACGGAGGTGAGG |
| human-XCR1-2 | CACAGGCAACAAGCAGGCGAACACCAGG |
| human-XCR1-3 | AGGAAGAAGATGCTGCTGTAGAGGCTGATGGAGAA |
| human-XCR1-4 | CTGGTTCTCACACGGCTGGCTCTGAAGGTC |
| human-XCR1-5 | GGAGAAGGCGAGGTTGCGGCAGATGAGC |
| human-XCR1-6 | GAGGCACAGGTTGAGGATGAAGATGTTGGTGAGG |
| human-XCR1-7 | CGAGGATGGAGGACAGGATGCTGGCTACC |
| human-XCR1-8 | CTCTCATACTTCACCAGGACCCACAGGACCAGG |
